# Patient‐Derived iPSC Neuronal Models Reveal Alzheimer’s Disease‐Associated Pathology and Provide a Platform for Drug Discovery

**DOI:** 10.1002/alz70861_108650

**Published:** 2025-12-23

**Authors:** Nadezhda Eckert, Elixirgen Scientific R&D

**Affiliations:** ^1^ Elixirgen Scientific, Baltimore, MD USA

## Abstract

**Background:**

Understanding the cellular mechanisms driving sporadic Alzheimer’s disease (AD) pathology is crucial for developing effective therapeutics. Induced pluripotent stem cell (iPSC)‐derived neurons and glia provide a patient‐specific, human‐relevant system for studying sporadic AD‐related phenotypes. We have developed an AD Panel consisting of differentiated neurons, microglia and astrocytes produced from iPSCs derived from AD patients, to systematically evaluate disease‐associated phenotypes and assess therapeutic interventions.

**Methods:**

We differentiated iPSCs from AD patients and healthy controls into excitatory neurons and glia and assessed key AD‐related phenotypes, including Aβ secretion and accumulation, tau hyperphosphorylation, synaptic integrity, and electrophysiological activity. Neurons were characterized using high‐resolution immunofluorescence imaging, ELISA, and MEA. Tri‐culture of neurons and glial cells was set up to evaluate a more complex model of sporadic AD.

**Results:**

AD patient‐derived neurons exhibited increased secretion and accumulation of amyloid peptides, an elevated Aβ42/Aβ40 ratio and increased pTau/Tau ratio in AD patient derived neurons. Mature AD neurons had altered electrophysiological activity, suggesting functional deficits. To evaluate the therapeutic potential of GSK3β and β‐secretase inhibitors (BACEi), we tested their effects on pTau and Aβ42 levels across multiple healthy and AD patient iPSC‐derived neuronal lines. Our data revealed a consistent reduction in pTau and Aβ42 levels in AD and healthy lines, while the response to both compounds was donor‐dependent, underscoring the importance of patient‐specific models for drug testing. We have found that coding variants in ABCA1 transporter may be responsible for sensitivity to the GSK3β inhibitor. MEA and ICC data suggests that older AD neurons have diminished activity due to loss of synaptic connections in our model.

**Conclusions:**

Our iPSC‐based AD Panel provides a robust, human‐relevant model for studying sporadic AD pathophysiology. The ability to recapitulate key AD hallmarks in patient‐derived neurons supports their use in target validation, biomarker identification, and personalized drug screening. This scalable model facilitates therapeutic discovery and precision medicine approaches in AD research.

